# Association between Brachial-Ankle pulse wave velocity and cardiac autonomic neuropathy in type 2 diabetes

**DOI:** 10.1186/1758-5996-6-82

**Published:** 2014-07-30

**Authors:** Nan Wu, Xiaoling Cai, Kuanping Ye, Yintao Li, Min He, Weiwei Zhao, Renming Hu

**Affiliations:** The Institute of Endocrinology and Diabetology, Huashan Hospital, Shanghai Medical College, Fudan University, 12 Middle Wulumuqi Road, Shanghai, China; The Emergency Department, Zhongshan Hospital, Shanghai Medical College, Fudan University, 180 Fenglin Road, Shanghai, China

**Keywords:** Cardiac autonomic neuropathy, Brachial-ankle pulse wave velocity, Type 2 diabetes

## Abstract

**Background:**

Cardiac autonomic neuropathy (CAN) is a common complication of type 2 diabetes mellitus (T2DM). Brachial-ankle pulse wave velocity (baPWV) is known to be a good surrogate marker of vascular damages. The goal of this study was to investigate the relationship between BaPWV and CAN in T2DM.

**Methods:**

A total of 148 patients who had no apparent history of cardiovascular condition were enrolled consecutively in this study. The correlation between increased baPWV and CAN was analyzed. CAN was evaluated by five standard cardiovascular reflex tests (CARTs) according to the Ewing's protocol: 1) heart rate variation during deep breathing, 2) heart rate response to standing, 3) Valsalva maneuver, 4) postural systolic blood pressure (BP) change, 5) Sustained handgrip test. CAN was defined as the presence of at least two abnormal tests.

**Results:**

The mean age of patients was 59.8 ± 7.8 years. The mean duration of diabetes was 6.0(2.0-11.0) years. The mean baPWV was 1665.5(1482.0-1940.0) cm/sec. Subjects with CAN were older and had high BMI, baPWV compared with those without CAN. The proportion of patients with diabetic peripheral neuropathy was higher in subjects with CAN. After adjusting for other confounding risk factors, baPWV (odds ratio = 8.496, 95% CI: 1.216-59.348; P = 0.031) remained as independent risk factors for CAN. The number of abnormal CARTs increased gradually with increasing baPWV (correlation coefficient =0.255, p = 0.002).

**Conclusion:**

Increased baPWV was significantly correlated with CAN in patients with type 2 diabetes.

## Introduction

Cardiovascular autonomic neuropathy (CAN) is a serious complication of diabetes, which was associated with cardiovascular morbidity and all-cause mortality in people with Type 2 diabetes mellitus [1, 2]. CAN was caused by damage to the autonomic nerve fibers that innervate the heart and blood vessels. It leads to dysfunctional heart-rate control and abnormal vascular dynamics, which will increase heart rate variability and decrease myocardial perfusion [3].

Pulse wave velocity (PWV) is known to be an indicator of arterial stiffness and a surrogate marker of vascular damages [4]. As brachial-ankle PWV (baPWV) is affected by vasomotor reflexes, it can reflect the statements of the aorta and peripheral arteries. The baPWV has been thought to have a great association with micro-angiopathic conditions and diabetes complications [5]. Previous reports indicated that baPWV is used as a severity index for subclinical atherosclerosis [6], and has been recommended as test for the assessment of target organ damage in arterial hypertension [7]. Aso et al. showed that baPWV was directly related to the frequencies of albuminuria, autonomic neuropathy, peripheral neuropathy, and retinopathy [5]. Bagherzadeh et al. reported that there exists a significant relationship between heart rate variability (HRV) and baPWV in diabetic patients [8].

This study used a retrospective analysis to investigate CAN in type 2 diabetes patients and the association between baPWV and cardiovascular autonomic reflex tests (CARTs).

## Method

### Study subject

We enrolled consecutive type 2 diabetes subjects (mean age. 59.8 years) who presented to outpatient department of endocrinology at Huashan Hospital between August 1, 2012 and February 30, 2013. Exclusion criteria included Type 1 diabetes characterized by a low C-peptide level (<0.3 ng/ml) and a history of diabetic ketoacidosis, other secondary causes of diabetes mellitus, malignancy, hepatic failure, acute metabolic complications, fatal arrhythmia, CVD such as acute coronary syndrome and previous myocardial infarction, cervical spondylosis, infectious polyneuritis, vasculitis, uremia, foot ulcers. Finally, 148 patients were enrolled in our study. Written informed consents were obtained from all participants and the study was approved by the local ethics committee of the Huashan Hospital.

### Data collection

A standardized questionnaire was designed to collect clinical information regarding the duration of diabetes, alcohol consumption, cigarette smoking and other health related variables. Body weight and height were measured with the participant wearing light clothing without shoes. Body mass index (BMI) was defined as weight in kilogram divided by square height in meter. Waist circumference was measured at the narrowest point between the lower limit of the ribcage and the iliac crest. Hip circumference was measured around the widest portion of the buttocks, with the tape parallel to the floor. Blood pressure (BP) was measured in the supine position and in a resting state using mercury sphygmomanometer with an appropriate cuff on the left arm and the average of the last two measurements was used.

A 12-hour overnight fasting venous blood sample was collected for measurement of plasma glucose, HbA1c, creatinine, total cholesterol, low-density lipoprotein cholesterol (LDL-C), high-density lipoprotein cholesterol (HDL-C), triglycerides (TG) using standard protocol. First morning urine was collected once a day for 3 consecutive days. Albumin-to-creatinine radio was calculated by dividing urinary albumin by urinary creatinine.

### Cardiac autonomic function test

Patients were instructed to refrain from alcohol, tobacco,caffeinated beverages and vigorous exercise during the 24 hours prior to the test. Before the test, the patients would fast at least 3 hours. Medications such as β-Blockers,antihistamines, antidepressants were withdrawn for 12 hours prior to the test. All the tests were performed by the same operator.

CAN was assessed by five standard cardiovascular reflex tests according to Ewing’s protocol [9]. These included heart rate (HR) responses to deep breathing (beat to beat variation), lying to standing HR responses (30:15 ratio), Valsalva maneuver, blood pressure(BP) responses to standing and sustained handgrip test. The heart rate response to deep breathing, standing, and the Valsalva maneuver were assessed automatically from electrocardiography recordings using the Sunrui evaluation system (ECG-2203B, Sunrui Co., Ltd., Guangzhou, China). The severity of CAN was quantified by summing the points obtained from each of the five tests, where each test scored with 0, 0.5, or 1 points depending on whether it yielded normal, borderline, or abnormal values, respectively. CAN was defined as the presence of at least two abnormal tests or autonomic neuropathy points ≥2 [10]. The CAN score was categorized as follows: CAN score 0 (total points 0), CAN score 1 (points 0.5 to 1.5), CAN score 2 (points 2 to 3), and CAN score 3 (points ≥3.5). CAN was considered absent, early, definite, or severe if the CAN scores were 0, 1, 2, or 3, respectively.

### Measurements of baPWV

VP-1000 Automatic Arteriosclerosis Measurement System (model BP-203 RPE-II, Colin Co, Ltd, Komaki, Japan) was used to measure BaPWV. The details of the measurement, validity, and reproducibility have been testified previously [11]. The device can record pulse waves by sensors in four cuffs, store data on the start point of each pulse wave in the right arm and both legs, record the time difference between transmission time to arm and transmission time to ankle as “transmission time”, calculate the transmission distance from the right arm to each ankle according to body height, and automatically compute the baPWV values by transmission time and transmission distance. Patients were instructed to have a 5 min rest in a supine position in a room with air-condition (24-26°C) before test. There was a significant positive correlation between left and right baPWV (r =0.953, P < 0.001). As a result, we used a mean of bilateral baPWV value during analysis.

### Evaluation of diabetic microvascular complications

Diabetic retinopathy (DR) was defined based on digital non-mydriatic fundus photography protocol which was modified from procedures used in previous studies [12]. The patients were given 5 min in a darkened room to allow dark adaptation. A trained photographer took a single undilated non-mydriatic digital photograph centred on the fovea of each fundus using a Canon CR6-45NM camera, repeated once only if necessary. The digitally stored fundus photographs were stored as bitmap images and viewed in a darkened room on CRT screens at a resolution of 1024 × 768 pixels. The digitally stored fundus images were graded by two endocrinologists and a retinal specialist. Retinopathy was classified as either absent or present.

The presence of diabetic nephropathy was defined as microalbuminuria (creatinine 30–300 mg/g) or overt albuminuria (creatinine > 300 mg/g). Urine albumin excretion was determined by measuring the urine albumin: creatinine ratio in spot urine samples [13].

Diabetic peripheral neuropathy (DPN) was assessed using Neuropathy Deficit Score (NDS) and Neuropathy Symptom Score (NSS). The diagnosis of DPN depends on both subjective symptoms and signs of neuropathy. We defined DPN as at least moderate signs with or without symptoms (NDS ≥6), or mild signs with moderate symptoms (NDS ≥3 and NSS ≥5) [14].

### Statistical analyses

Analyses were performed using IBM SPSS 19.0 for Windows (SPSS Inc., an IBM company, USA). Numerical variables with normal distribution are expressed as means ± SD, and reported as median (interquartile range) and log transformed to approximate normality before analysis otherwise. Pearson correlational analysis was used to examine the relationships between baPWV and other metabolic variables in Type 2 diabetes. The independent-samples t test or chi-squared tests were used to compare the differences in clinical and biochemical characteristics between patients with and those without CAN. Multivariate logistic regression analyses were performed to estimate the contribution of baPWV to cardiac autonomic neuropathy using the odds ratio and 95% CI. P values < 0.05 were considered statistical significant.

## Results

### General characteristics of participants

The clinical and biochemical characteristics of the study population are shown in Table 1. A total number of 148 Chinese (80 men and 68 women) were included. Mean age, BMI and the duration of diabetes were 59.8 ± 7.8 years, 24.4 ± 3.4 kg/m2, and 7.2 ± 5.8 years, respectively. Mean baPWV was 1665.5(1482.0-1940.0) cm/sec. Prevalence rates of hypertension, diabetic retinopathy, diabetic peripheral neuropathy and diabetic nephropathy were 54.7, 16.9, 31.1 and 22.2%, respectively. Prevalence of hypertension was 54.7% and the information of anti-hypertensives use was shown in Table 1. Furthermore, prevalences of early, definite and severe cardiac autonomic neuropathy were 39.2, 52.7 and 7.4%, respectively.

**Table 1:** **Clinical and biochemical characteristics of participants**

Characteristic	N = 148
cardiovascular autonomic neuropathy(%)	78(52.7)
Age(years)	59.8 ± 7.8
Male,n(%)	80(54.0)
Current smoker, n (%)	43(29.0)
Alcohol consumption, n (%)	37(25.0)
Hypertension, n (%)	81(54.7)
Use of β-Blockers	34(22.9)
Use of ACEI/ARB	67(45.3)
Use of CCB	48(32.4)
Other antihypertensives	14(9.5)
Express as median(years)	6.0(2.0-11.0)
BMI(kg/m^2^)	24.4 ± 3.4
Waist-to-hip ratio	0.94(0.90-0.99)
systolic blood pressure(mmHg)	134.2 ± 18.1
diastolic blood pressure(mmHg)	78.3 ± 10.6
HbA1c(%)	8.0 ± 1.9
fasting glucose(mmol/l)	7.1(5.8-9.2)
Creatinine (μmol/L)	62.0(53.0-78.0)
eGFR( ml/min/1.73 m^2^)	107.0 ± 32.8
total cholesterol(mmol/l)	4.89 ± 1.52
Triglyceride(mmol/l)	1.61(1.05-2.34)
LDL cholesterol(mmol/l)	2.84(2.29-3.60)
HDL cholesterol(mmol/l)	1.09(0.93-1.33)
ACR(mg/g)	12.02(7.18-19.54)
baPWV(cm/sec)	1665.5(1482.0-1940.0)
Diabetic retinopathy, n (%)	25(16.9)
Diabetic peripheral neuropathy, n (%)	46(31.1)
NSS score	5.0(2.0-6.0)
NDS score	4.0(2.0-5.0)
Diabetic nephropathy, n (%)	33(22.2)

BMI, body mass index; eGFR, modification of diet in renal disease study-glomerular filtration rate; ACR, albumin-to-creatinine ratio;ACEI/ARB, angiotensin converting enzyme inhibitors/Angiotensin II receptor blockers; CCB, Calcium channel blocker; NSS, neuropathy symptom score; NDS, neuropathy disability score.

Data are means SD, median (25-75%), or number (percent).

### Correlation between baPWV and other clinical variables

Table 2 shows the relationships between baPWV and other clinical variables. baPWV was significantly correlated with age (r = 0.404, p < 0.001) and systolic blood pressure (r = 0.654, p < 0.001). Our results indicated that baPWV was not associated with duration of diabetes, BMI, Waist-to-hip ratio, HbA1c fasting glucose, creatinine, eGFR and lipid variables.

**Table 2:** **Correlation between baPWV and other clinical variables**

Variable	Correlation coefficients	P
Age	0.404	0.001
Duration of diabetes*	0.141	0.095
BMI	0.124	0.134
Waist-to-hip ratio*	0.081	0.327
systolic blood pressure	0.654	0.001
diastolic blood pressure(mmHg)	0.327	0.001
HbA1c	-0.010	0.900
fasting glucose*	0.061	0.477
Creatinine*	0.020	0.832
eGFR	-0.128	0.122
total cholesterol	-0.038	0.694
Triglyceride*	0.151	0.116
LDL cholesterol*	-0.028	0.779
HDL cholesterol*	-0.073	0.459
ACR(mg/g)	-0.028	0.781

Data are shown as Pearson correlation coefficients (r).

baPWV was log-transformed when statistics were applied.

*Denotes the variables that were log-transformed when statistics were applied.

### Characteristics of participants according to the presence of cardiac autonomic neuropathy

Shown in Table 3, subjects with CAN were older and had larger BMI(25.0 ± 3.6 vs. 23.9 ± 3.1, p = 0.042) and higher baPWV(1722.0(1493.9-2040.8) vs. 1584.0(1446.1-1812.63), p = 0.011). The prevalence of diabetic peripheral neuropathy was higher (38.5% vs 22.9%, p = 0.041) and NSS score was higher in subjects with CAN. Other microvascular complications including diabetic retinopathy and diabetic peripheral neuropathy did not significantly differ between the two groups. Duration of diabetes, the presence of hypertension and other biochemical findings did not differ significantly according to the presence of CAN.

**Table 3:** **Comparison of clinical characteristics according the presence of cardiac autonomic neuropathy**

Variable	without CAN (n = 70)	with CAN (n = 78)	P value
Age(years)	57.6 ± 7.6	61.8 ± 7.4	0.001
Male,n(%)	41(58.6)	39(50.0)	0.296
Current smoker, n (%)	17(24.3)	26(33.3)	0.226
Alcohol consumption, n (%)	15(21.4)	22(28.2)	0.342
Hypertension, n (%)	35(50.0)	46(59.0)	0.273
Use of β-Blockers	16(22.9)	18(23.1)	0.856
Use of ACEI/ARB	32(45.7)	35(44.9)	0.746
Use of CCB	22(31.4)	26(33.3)	0.246
Other antihypertensives	8(11.4)	6(7.7)	0.168
Duration of diabetes(years)*	6.0(2.0-10.3)	6.0(2.0-11.0)	0.815
BMI(kg/m^2^)	23.9 ± 3.1	25.0 ± 3.6	0.042
Waist-to-hip ratio*	0.94(0.88-0.99)	0.94(0.90-1.00)	0.849
systolic blood pressure(mmHg)	132.2 ± 16.4	136.0 ± 19.4	0.207
diastolic blood pressure(mmHg)	78.7 ± 11.0	78.0 ± 10.3	0.691
HbA1c(%)	7.9 ± 1.7	8.1 ± 2.0	0.613
fasting glucose(mmol/l)*	7.1(6.0-9.1)	6.7(5.8-9.3)	0.750
Creatinine (μmol/L)*	64.0(52.0-78.0)	59.0(53.0-79.5)	0.519
eGFR( ml/min/1.73 m^2^)	107.8 ± 25.4	103.0 ± 31.1	0.303
total cholesterol(mmol/l)	4.83 ± 1.92	4.94 ± 1.16	0.709
Triglyceride(mmol/l)*	1.54(0.99-2.69)	1.75(1.10-2.33)	0.510
LDL cholesterol(mmol/l)*	2.84(2.11-3.60)	2.83(2.37-3.60)	0.632
HDL cholesterol(mmol/l)*	1.10(0.92-1.30)	1.09(0.94-1.36)	0.693
ACR(mg/g)	10.90(6.87-24.72)	15.41(7.60-23.67)	0.472
baPWV(cm/sec)*	1584.0(1446.1-1812.63)	1722.0(1493.9-2040.8)	0.011
Diabetic retinopathy, n (%)	10(14.3)	15(19.2)	0.423
Diabetic peripheral neuropathy, n (%)	16(22.9)	30(38.5)	0.041
NSS score*	3.0(2.0-5.0)	5.0(3.0-6.0)	0.036
NDS score*	3.0(2.0-4.0)	4.0(2.0-5.0)	0.065
Diabetic nephropathy, n (%)	16(22.9)	17(21.8)	0.877

BMI, body mass index; eGFR, modification of diet in renal disease study-glomerular filtration rate.

Data are means SD, median (25-75%), or number (percent).

*Denotes the variables that were log-transformed when statistics were applied.

### baPWV and CAN in patients with Type 2 diabetes

Age greater than 60 years old, BMI and baPWV were significant risk factors for CAN (Model A in Table 4); however, adjusted for age, gender, duration of diabetes, BMI, HbA1c, systolic blood pressure, the presence of diabetic retinopathy, the independent risk factors remained baPWV (odds ratio = 8.496, 95% CI 1.216–59.348; P = 0.031).

**Table 4:** **Relationships between multiple risk factors and cardiovascular autonomic neuropathy**

Variable	Model A		Model B	
	Adjusted odds ratio (95% CI)	P	Adjusted odds ratio (95% CI)	P
Age > 60 years, no vs yes	2.247(1.160-4.349)	**0.016**	2.837(1.365-5.897)	**0.005**
Gender(male)	1.513(0.776-2.951)	0.225	1.692(0.840-3.406)	0.141
Duration of diabetes(per decade)	0.848(0.474-1.517)	0.578	0.832(0.454-1.523)	0.550
BMI(per kg/m2)	0.888(0.800-0.985)	0.024	0.888(0.798-0.988)	0.029
Waist-to-hip ratio*	2.205(0.014-353.823)	0.760	2.119(0.320-14.033)	0.436
systolic blood pressure(per 10 mmHg)	1.008(0.989-1.027)	0.391	1.017(0.996-1.038)	0.108
diastolic blood pressure(per 10 mmHg)	0.997(0.966-1.028)	0.840	0.977(0.919-1.039)	0.457
Current smoker, no vs yes	1.660(0.792-3.479)	0.179	1.331(0.599-2.955)	0.482
Alcohol consumption, no vs yes	1.521(0.702-3.292)	0.287	1.474(0.660-3.293)	0.344
Hypertension, no vs yes	1.262(0.645-2.468)	0.497	1.563(0.767-3.183)	0.219
HbA1c(per 1%)	1.115(0.929-1.338)	0.244	1.091(0.905-1.314)	0.550
fasting glucose(per 1 mmol/l)*	1.020(0.922-1.128)	0.700	0.594(0.176-2.007)	0.401
Creatinine (per 1 μmol/L)*	0.954(0.230-3.950)	0.948	1.119(0.256-4.882)	0.881
eGFR( per 1 ml/min/1.73 m2)	0.999(0.987-1.011)	0.874	0.995(0.982-1.008)	0.465
total cholesterol(per 1 mmol/l)	1.046(0.807-1.355)	0.735	1.024(0.778-1.349)	0.864
Triglyceride(per 1 mmol/l)*	0.823(0.462-1.469)	0.511	0.969(0.524-1.795)	0.921
LDL cholesterol(per 1 mmol/l)*	1.405(0.426-4.634)	0.576	1.470(0.423-5.113)	0.545
HDL cholesterol(per 1 mmol/l)*	0.740(0.242-2.267)	0.598	0.632(0.196-2.034)	0.442
ACR(mg/g)	1.000(0.999-1.000)	0.574	1.000(0.999-1.000)	0.538
baPWV(cm/sec)*	6.117(1.032-36.245)	0.046	8.496(1.216-59.348)	0.031
Diabetic retinopathy, no vs yes	1.431(0.586-3.493)	0.431	1.589(0.634-3.984)	0.323
Diabetic peripheral neuropathy, no vs yes	2.071(0.994-4.315)	0.052	2.672(1.155-6.183)	0.022
Diabetic nephropathy, no vs yes	0.940(0.426-2.072)	0.878	1.087(0.459-2.575)	0.849

Data were obtained from a multivariate regression model.

Model A, adjusted for age; Model B, adjusted for age, gender, smoking, drinking, duration of diabetes, BMI, HbA1c, systolic blood pressure, the presence of diabetic retinopathy.

*Denotes the variables that were log-transformed when statistics were applied.

**Figure 1:**
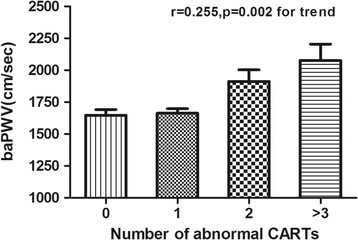
**The levels baPWV according to the number of abnormal CARTs.** Data are shown as means ± SE after adjustment for age (per decade), gender, smoking, duration of diabetes; P =0.002 for trend.

Remarkably, baPWV increased gradually with increasing number of abnormal CARTs (Figure 1). The mean values (SE) of baPWV significantly increased for those with one to above three CARTs were 1647 (44.76), 1663 (36.82), 1911 (91.95) and 2076 (127.40) cm/sec after adjustment for age (per decade), gender, smoking, duration of diabetes. As shown in Table 5, baPWV was significantly higher in patients with abnormal heart rate variation during deep breathing and HR response to standing tests, compared to patients with normal and borderline result tests while there was no such difference observed regarding the valsalva maneuver , postural BP change and sustained handgrip test. After adjustment for age, gender and duration of diabetes, the difference in baPWV between patients with normal and abnormal tests remained significant for heart rate variation during deep breathing (p = 0.002) and HR response to standing tests (*p =* 0*.*046).

**Table 5:** **Mean baPWV value in patients with normal, borderline, and abnormal autonomic nervous function tests**

		Normal result	Borderline result	Abnormal result	***P***for trend
baPWV(cm/sec)*	HR variation during deep breathing	1537.50(1392.88-1863.00)	1649.25(1480.75-1815.50)	1728.25(1524.00-2029.38)##	0.002
	HR response to standing	1623.75(1444.38-1890.00)	1757.25(1552.75-2012.75)	1825.00(1509.88-2020.63)#	0.046
	Valsalva maneuver	1666.25(1459.88-1945.75)	1642.00(1512.25-2006.50)	1669.50(1445.25-1934.75)	0.392
	postural BP change	1651.75(1486.88-1937.00)	1661.25(1426.50-1983.50)	1775.86(1568.67-2043.00)	0.471
	Sustained handgrip test	1656.00(1500.13-1947.50)	1540.75(1329.37-1939.63)	1777.25(1509.88-1943.38)	0.835

HR, heart rate; BP, blood pressure.

*Denotes the variables that were log-transformed when statistics were applied.

#P < 0.05, ##P < 0.01, for comparisons of baPWV values between abnormal, borderline and normal test.

## Discussion

The present study demonstrates that baPWV was positively associated with the prevalence of cardiac autonomic neuropathy in Type 2 diabetes. These relationships persisted in the multivariate model after adjustment for known risk factors. baPWV was known as a good surrogate markers of clinical atherosclerosis and vascular dysfunction [4]. Our results suggested that CAN might be an important factor of clinical atherosclerosis in T2DM. This finding of the association between CAN and baPWV (a good sign of vascular dysfunction) may help to explain the excess cardiovascular disease mortality seen in those with T2DM subjects with CAN.

The main pathological findings for CAN are damages to the autonomic nerve fibers that innervate the heart and blood vessels and this resulted in abnormalities in heart rate control and vascular dynamics [3]. Previous studies have reported that CAN was associated with accelerated atherosclerosis, represented as carotid intima media thickness (CIMT) and plaques, independent of the traditional cardiovascular risk factors in T2DM [15, 16]. Therefore, the presence of CAN should be considered and accessed when patients are in the early stage of diabetes, rather than after the development of clinical cardiovascular disease.

Pulse wave velocity describes the rigidity of the arterial wall, and is an indicator of atherosclerosis irrespective of classical cardiovascular risk factors and ethnicity [17]. Many prospective studies showed that PWV had more advantages in reflecting the risk of cardiovascular disease than the blood pressure and had a strong relationship with cardiovascular events and all-cause mortality [18]. As a simpler noninvasive method, brachial-ankle PWV (baPWV) integrates the mechanical properties from both the central and peripheral arteries. It has a high correlation with traditional carotid-femoral PWV (cfPWV), and it is suitable for general population studies [6]. Previous studies have indicated that baPWV was not only directly related to macrovascular complications but also micro-angiopathic conditions and diabetes peripheral neuropathy. Yokoyama et al. reported that baPWV, retinopathy, age, and glycated hemoglobin were independent risk factors in DPN and the associated autonomic neuropathy [19]. Byung et al. reported that increased baPWV was significantly correlated with peripheral neuropathy in patients with type 2 diabetes which was diagnosed by total symptom score (TSS) and abnormal neurological assessment [20]. As to the relationship between baPWV and CAN, a recent study [8] pointed out that the association between PWV and CAN was independently significant in diabetes, which used heart rate variability (HRV) as an index for CAN. Our study further confirmed the association between CAN and baPWV using CARTs as diagnosis criterion for CAN. In a univariate analysis of the risk factors for CAN, the odds ratio for baPWV was 6.117(95% CI, 1.032-36.245; *P* = 0.046), which is a meaningful risk factor for CAN. A multivariate logistic regression analysis was performed to adjust for confounding variables and the adjusted odds ratio was 8.496(95% CI, 1.216-59.348; P = 0.031), which indicates that baPWV might be an independent risk factor for CAN.

Previous researches pointed out the primary factors affecting baPWV are age, systolic blood pressure, and gender [21]. In a Korean study on type 2 diabetes patients, baPWV was significantly correlated with mean blood pressure, heart rate, age, urine albumin/creatinine ratio [22]. In our study, age, systolic blood pressure and diastolic blood pressure were positive related with baPWV. Duration of diabetes, BMI, glycated hemoglobin, lipid levels were not correlated with baPWV. To our surprise, urine albumin/creatinine ratio which have been proved to be significantly associated with arteriosclerosis and autonomic function [23, 24] was not related with baPWV in our study. That may be because 85.0% of our patients had normal urine albumin/creatinine ratio which weaken the link between urine albumin/creatinine ratio and baPWV.

According to our results, baPWV was significantly higher in patients with abnormal heart rate variation during deep breathing and HR response to standing tests. Both heart rate response to deep breathing (deep breathing test) and heart rate response to standing (standing test) which concerns heart rate variation), reflect mainly parasympathetic activity of the heart innervation although the sympathetic nervous system may somewhat affect these reactions [3, 25]. But heart rate variation during the Valsalva maneuver and sustained handgrip test is mediated by combined effect of parasympathetic and sympathetic nerve fibres [3, 26]. Our study showed that the increase of baPWV better reflected the damage of parasympathetic nerve fibres and for those CARTs who have a dual innervation (parasympathetic and sympathetic nervous system), the change of baPWV was insensitive. Our study also demonstrated that the number of abnormal CARTs increased gradually with increasing baPWV. In other word, the surge of baPWV is a comprehensive reflection of injuries of the sympathetic nervous system and the parasympathetic nervous system. The association between PWV and cardiac autonomic function also has been examined in a small study in healthy individuals [27]. Consistent with our results, heart rate response to deep breathing showed moderate correlations with PWV. CARTs mainly characterizing sympathetic function had no correlation with aortic stiffness parameters including PWV.

The relationship between the decrease of large arteries elasticity and the dysfunction of autonomic nervous system in type 2 diabetes is not fully understood. It’s not clear whether arterial stiffness produce dysfunction of the cardiac autonomic nervous system or CAN induce stiffness of the large arteries. Some study suggested that dysfunction of the cardiovascular autonomic system may influence the elasticity of the arterial wall by affecting the vascular tone of large arteries [28]. Other studies indicated that tachycardia which induced by CAN is main reason for arterial stiffness. Indeed, increases in heart rate lead to arterial stiffness independently of changes in activity of the autonomic nervous system [29]. The present analysis showed that there was correlation between heart rate response to deep breathing, standing and baPWV, which partly showed the association between parasympathetic dysfunction and arterial stiffness.

The limitations of our study resulted from retrospective cross-sectional design, which did not allow drawing conclusions about any causal relation between CAN and baPWV. We also could not assess how the changes in both CARTs and baPWV through time, as well as the changes in the serum glucose levels and glycosated hemoglobin, affect each other. Secondly, our study was conducted in outpatient department of endocrinology and we formulated strict exclusion criteria. It largely decreased the number of patients in our study. CART tests required patients to actively cooperate with us and the entire check need relatively long time. Many elderly people and patients with severe diabetic complication refused to participate in our study or dropped out from the study. So the participants in our study were younger and had better health status, which means the conclusion of our study may not be applied to other diabetes patients. Moreover, the effects of the type of diabetes treatment and other prescribed medications on CAN and PWV need to be investigated in future studies.

In conclusion, CAN, particularly the injury of parasympathetic activity of the heart, as expressed by abnormal heart rate variation during deep breathing and standing, correlates strongly with baPWV in patients with type 2 diabetes. Most interestingly, this finding was observed in a population without apparent history of cardiovascular condition.

## Abbreviations

CAN: Cardiac autonomic neuropathy
baPWV: Brachial-ankle pulse wave velocity
CARTs: Cardiovascular reflex tests
BP: Blood pressure
T2DM: Type 2 diabetes mellitus
HRV: Heart rate variability
HR: Heart rate
DR: Diabetic retinopathy
DPN: Diabetic peripheral neuropathy
BMI: Body mass index
eGFR: Modification of diet in renal disease study-glomerular filtration rate
ACR: Albumin-to-creatinine ratio.
